# Use of methylphenidate in alzheimer’s dementia: Effect on apathy

**DOI:** 10.1192/j.eurpsy.2021.1135

**Published:** 2021-08-13

**Authors:** R. Borralho, C. Oliveira

**Affiliations:** 1 Psiquiatria Geral E Transcultural, Centro Hospitalar Psiquiátrico de Lisboa, Lisboa, Portugal; 2 Clínica 3, Centro Hospitalar Psiquiátrico de Lisboa, Lisboa, Portugal

**Keywords:** dementia, Alzheimer, methylphenidate, apathy

## Abstract

**Introduction:**

Alzheimer’s Disease (AD) is associated with neuropsychiatric symptoms such as agitation depression and apathy. It has been proposed that the pathophysiology of apathy, that is defined as quantitative reduction in goal-directed activity compared with previous functioning, in AD is associated with degeneration of prefrontal cortex and dysfunction of dopamine and norepinephrine neurons in the brain. Methylphenidate (MPH) is a dopamine and norepinephrine reuptake inhibitor and its action increase the availability of these neurotransmitters in the extracellular space of striatum and prefrontal cortex. Over the past decade there has been an effort to study the benefit of the use of MPH for treatment of apathy in patients with Alzheimer’s dementia.

**Objectives:**

Study the benefit of methylphenidate in the treatment of apathy in AD.

**Methods:**

Basic literature review collecting data from PubMed (2010-2020) using the words “Methylphenidate”, “Apathy”, “Alzheimer”, “Dementia”.

**Results:**

Clinical trials using 10 to 20mg of MPH per day, for 6 weeks, demonstrated a mitigation in apathy symptoms in one third of patients, with good tolerability. Another clinical trial using the same dosage, for 12 weeks, led to improvement in cognition, functional status, depression and caregiver burden.

**Conclusions:**

New clinical trials with larger groups of patients over a longer period are needed to consolidate the existing results. Although there are still many questions concerning the usefulness of methylphenidate in this population that need to be answered, methylphenidate might be an option to deal with one of the most prevalent neuropsychiatric symptoms, apathy, in some AD patients.

